# Open-Source web-based geographical information system for health exposure assessment

**DOI:** 10.1186/1476-072X-11-2

**Published:** 2012-01-10

**Authors:** Barry Evans, Clive E Sabel

**Affiliations:** 1 Geography, College of Life and Environmental Sciences, University of Exeter, Amory Building, Rennes Drive, Exeter, EX4 4RJ, UK; 2 European Centre for Environment and Human Health, Peninsula College of Medicine and Dentistry, Knowledge Spa, Royal Cornwall Hospital, Truro, TR1 3HD, UK

**Keywords:** Health and Environment spatial data, decision support system, GIS, Open source, web-based

## Abstract

This paper presents the design and development of an open source web-based Geographical Information System allowing users to visualise, customise and interact with spatial data within their web browser. The developed application shows that by using solely Open Source software it was possible to develop a customisable web based GIS application that provides functions necessary to convey health and environmental data to experts and non-experts alike without the requirement of proprietary software.

## Introduction

Using maps to visualise data can enable quicker interpretation of complex geographical phenomena [1], identify patterns, and aid in planning, resource allocations for policy and decision making [2]. Mapping, in the context of the Environment and Health sub-discipline, provides a visual assessment for investigating the spatial distribution of a disease and potential associations and underlying causes [3]. Developments in Geographical Information Systems (GIS) have now made the mapping of this information commonplace and are used in a large range of applications. Within the environment and health fields, recent applications using GIS have been used in projects such as identifying regions at risk to malaria [4], monitoring effects of air pollution on asthmatics [5] and defining an "*Index of Relative Wellbeing*" for an area from census data [6].

The research presented here, reports on results from the European Union (EU) FP6 funded Health and Environment Integrated Methodology and Toolbox for Scenario Assessment (HEIMTSA) project. The project's overall goal was to support the European Union's (EU's) Environment and Health Action Plan (EHAP) by extending health impact assessment (HIA) coupled with cost benefit analysis (CBA) methods and tools to evaluate the impacts policy scenarios have (at a European level) on the environment and human health. HEIMTSA was structured around loosely coupled modules that deal with pollutants and their media using the full chain (impact pathway) approach. The modelling tools and framework that were developed within this project was focussed on exploring:

• Emissions to environmental media ('stressor identification'), derived from sector scenarios in transport, energy, agriculture, industry, households and waste treatment and disposal, that are combined and harmonized to result in consistent scenarios for all relevant stressors for the whole of Europe;

• Human exposures (e.g. outdoor and indoor air pollution, water, noise, odour, metals, dioxins) by multiple routes, estimated, using new methods (exposure scenarios and probabilistic modelling), including consumer exposure;

• Health risk functions, derived, with new methods for: effects of combined exposures; estimating background rates; and mapping health impacts, to aid in communication of results; and

• Monetary valuation including a review of methods for valuating children's health, developing values for relevant health endpoints, and extending the valuation paradigm to include altruism.

These methodological topics were integrated within an online modelling toolbox where users could request models to be implemented based on specific inputs and later download the results. A drawback of this approach was that it required the user to know how to interpret the results and also have appropriate software to visualise their data.

With communication and data sharing defined as underpinning elements that aid in dealing with cross border health and environmental issues [7], HEIMTSA set out to improve communication by enabling the access and viewing of (spatial) data and information easily online to expert and non expert users alike. The goal of this part of the project was to allow users anywhere in the world the ability to look at and interact with their model results in an online environment and share their findings with others. Commercial software which is designed to use this type of data online already exists but it can often come with a high initial set up cost, ongoing licensing fees and require some degree of technical knowledge to operate. If HEIMTSA was to overcome these problems it needed to provide a way to allow users to visualise their data freely online and be simple to use. For this an open source web GIS platform was chosen that would enable users to access, visualise and interact with their data online within a web browser.

Online publishing of georeferenced data is not new; spatial data has been published online since the early 1990s, shortly after the web became publicly available. The first internet mapping solutions were simple, only able to produce maps as static image files for example a Graphics Interchange Format (GIF), then shortly after, in the mid 1990s with the appearance of JavaScript (which allowed users to execute client side requests), interactive services became available such as pan, zoom, and query. The rapid growth in popularity of the internet, emerging opportunities to publish georeferenced data and the growing public interest in accessing spatial information, combined to spawn a large number of web mapping applications such as MapGuide, Mapquest, MultiMap, and ArcIMS; some commercial, and others, free and open to the general public [8]. The commercial software is predominantly "closed source" which means the code cannot be accessed or modified and (as mentioned earlier) it may, in some cases, come at a relatively high price with ongoing licensing fees to maintain use. These systems are thus often priced out of the reach of the resource-constrained areas of public health, particularly within developing nations, which leads them into seeking open source alternatives [9].

The rapid development of internet technology and emerging number of web GIS applications created the need to standardise implementation of the web map server interface. The Open Geospatial Consortium (OGC) first published a specification in 1999 [10]; which after revision became the Web Map Server Interface Implementation Specification (version 1.0) released in 2000. Further revisions of this specification to standardise map server implementations have been made since up to the current operating version [11]. Recently established web mapping systems can provide one or more of the OGC service types:

1. A Web Map Service (WMS) renders geo-referenced "information" as digital image files providing static maps to clients [11];

2. Web Feature Service (WFS) (unlike WMS) returns with actual feature (vector layers). Therefore rendering the user requested data - dependent on file size - takes noticeably longer than WMS map images. WFS also allows clients to edit stored layers [12]; and

3. Web Coverage Service (WCS) supports electronic retrieval of geospatial data as "coverages" (digital geospatial information representing space-varying phenomena). The WCS returns the original geo-referenced data together with its associated attribute data thus providing opportunities for data exploration and interpretation [13].

In recent years there have been many systems utilising alternative open source web GIS methods. Previous developments in open source include a GIS Enabled Cancer Atlas as a means of providing users the ability to visualise and interact with data relating to the distribution of cancer across the state of Pennsylvania [14]; a mapping system that looked at sharing biodiversity information [15]; a support tool for assessing the implementation of cross-border and global health spatial information systems (CBHSIS) across the US-Mexico border [16], and more recently a flash based web GIS solution was developed in Australia that enables users to map their own data online via importing tables [17].

There are some underlying difficulties associated with developing in open source when compared to closed source products bought "off the shelf". Open source applications often require greater amounts of time and computational expertise [18] and the professional support available often depends on the maturity of software and the size of the user community [19]. These difficulties aside, however, it is the low cost and customisability of open source that still makes it an appealing alternative for many users.

This paper specifically documents the design, development and implementation of a new web based GIS/spatial visualisation tool built from a combination of open source software packages as a support tool for the health and environment sectors. The tool provides the ability to access model results online and visually explore data in web browsers without the need of additional software. The advantage of developing an entirely new system is that it can be designed to: closely fit the task, be user friendly, and require no expert knowledge of GIS to operate.

## Methodology

The online modelling tools previously produced as part of the HEIMTSA work outputted their results in a textual/tabular format which were downloaded by the user and visualised utilising their own software. The issue with this approach was that it limited the distribution of information as it required the user to have the appropriate software to visualise the data and also have the skill set to do so. To solve this issue, a rudimentary GIS was proposed to allow users the ability to spatially visualise and interact with their data solely within a web environment.

The development of the web GIS tool was done via a bottom-up approach and was sub-divided in to four main design stages:

1. System requirements

a. Essential features

b. Additional features

2. Basic system design

3. Software selection

4. Development.

### System requirements

Due to the short time frame for the development of the web GIS system the overall development was broken up into two categories consisting of essential (high priority) and additional (low priority) features (Table 1). The "essential" requirements of the system were defined as those necessary for the user to be able to easily visualise, interact and query data outputted by the external modelling tool; whereas the "additional" features may not be needed in what is regarded as "standard" use of the visualisation tool but could prove useful for further analysis.

**Table 1:** Visualisation tool features

Essential	Additional
Link to Model outputs	View uncertainty (duel mapping)

Visualise data	Generate chart information

Style data on the fly	

Get additional information by selecting/querying the data	

The development of the visualisation tool was prioritised by determining which features are needed (essential) and which would be good to have (additional). The "essential" features are those deemed necessary for basic visualisation and querying of data. The "additional" allows for further querying and analysis of data.

To make the system user-friendly it was designed so that a user with little to no knowledge of GIS would be able to visualise their data. The simplest scenario was that via the user simply clicking on one button they could visualise their data spatially and immediately gain some insight into their results, using an exploratory spatial data analysis paradigm.

### Basic system design

**Figure 1:**
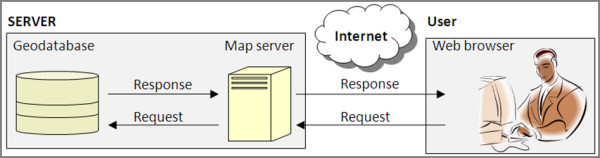
**Basic web GIS setup**. A basic web GIS is comprised of three parts; a geodatabase, mapserver and web viewer.

A basic web GIS system normally consists of three key parts; a geodatabase, map server, and web viewer (Figure 1). Our basic design criteria meant that: the software used must be built using solely open source software; no additional software would be required on the user side (there are some open source GIS systems that require a user to download desktop interfaces (for example GRASS [20]; and uDIG [21]); and the web viewer should be simple to operate so the user can easily interact with their data.

### Software selection

For the geodatabase, two popular open source database management systems (DBMS) were considered: MySQL and PostgreSQL (coupled with PostGIS). Both of these DBMS have very powerful spatial support systems incorporated into them but in terms of spatial functionality, PostgreSQL-PostGIS appeared to have a larger range [22] thus allowing for greater potential of expansion of the developed web GIS software in the future. This potential, coupled with PostgreSQL-PostGIS being chosen by many of recent web GIS solutions ([23,14,15,24], and [25]) lead the authors to choose this geodatabase for this project.

The map server makes it possible to access and display spatially enabled content of the geodatabase and enable querying and analysis of the displayed data [26]. It works by storing data as tables in the database that can be later viewed as layers of a map [14]. As with the geodatabase solutions, there were two open source map servers considered: MapServer and GeoServer. Reviewing previous studies ([27] and [24]) and discussion forums ([28] and [29]) it was deemed that the functionality between these two packages was quite similar and both packages would provide the level of functionality required for the project. GeoServer was subsequently chosen due to the developer's preference of its web administrator tool to aid in the testing and development of the web GIS system.

The primary design criterion for the web viewer was that no additional software would be required by the user; therefore spatial data could be viewed by the user solely within a standard web browser. The OpenLayers javascripting libraries [30], provides useful codes and commands making it possible for the user to visualise and interact with spatial data within a web browser. In addition OpenLayers; Ext and GeoExt javascript libraries ([31] and [32]) have also been chosen as these allow for ease of development of a professional looking web interface that resembles desktop applications via the use of familiar windows, combo boxes, and command buttons [8].

### Development

Once the basic components were selected, the development and testing of the system took place. Three things were considered during the development process that related to the "essential" and "additional" requirements of the software, namely the ability to:

1. Interpret model outputs from the pre-existing toolbox;

2. Visualise and interact with data; and

3. Incorporate additional features.

The overall system development is discussed in greater detail in the following section.

## Results

**Figure 2:**
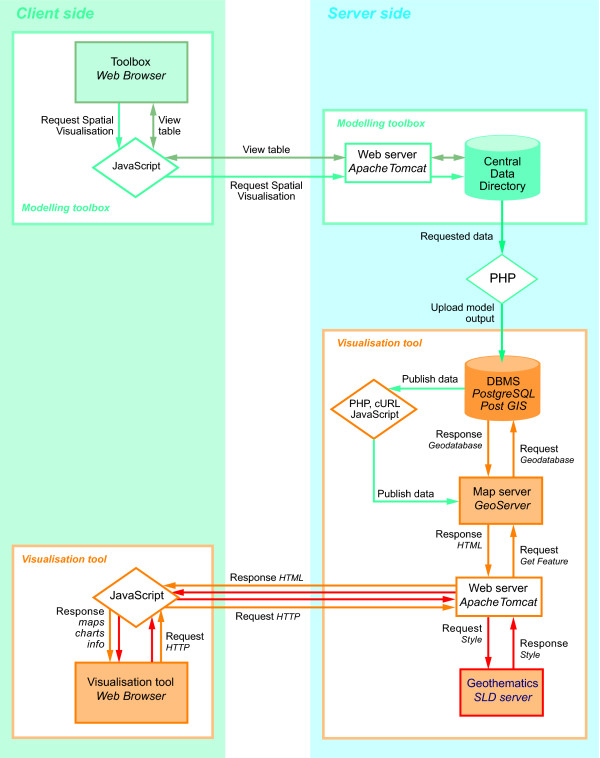
**System flow diagram**. The finalised system is divided up into two sides, a client (user) and a server side. There are three possible pathways that are used in the system one for uploading data to geodatabase and publishing data (green paths), one for visualising and querying data (orange paths), and finally one for styling the data (red paths).

In this section the design of the finalised web GIS system and its operation are discussed. The overall system design, shown in Figure 2, is divided into two sides; the user (client) side, and the server side. The flow of information within the system follows three distinct pathways:

1. Publish new model outputs (green path);

2. Display and Interact with data (orange path); and

3. Style data on the fly (red path)

Each of these pathways represents an essential process that takes place in the system. The basic principle is that all new data must go through the green path for preparation; the orange path for visualisation and interaction and the red path for styling and customisation. The following section details the functions of each process/pathway.

### Publish new model outputs (green path)

To visualise any health and/or environment model output in the web viewer it must first be assigned geographical/spatial attributes and published within the map server (GeoServer). The outputs from the modelling tool tested in this paper are in a comma separated variable (CSV) format and come in two versions; one being point data and the other country level polygon data. The point data's resolution is determined by the model but consists of unprojected latitude and longitude values. The country level data only has "country code" information that relates to its location and does not possess any geometric information at this stage. Within the geodatabase is a pre-existing country level polygon dataset; this dataset already contains spatial and geometric information and shares the same "country code" key field for each country polygon as those used in the outputs from the modelling tool. Using the "country codes" as a relational join field, the two datasets can be joined together in the geodatabase to enable spatial visualisation.

Once the data has been uploaded to the geodatabase and has spatial attributes, it needs to be "published" within GeoServer. The publishing of data allows GeoServer the ability to interpret the data and render it at a later stage within the web viewer. The information required by GeoServer includes the bounding box of the data (the extent), the projection of the data, and a default style which is to be applied. Publishing is commonly done via the web administrator tool within GeoServer. This, however, is impractical in our online application as it would then require the user to access the web admin tool, reference the data in the geodatabase and define parameters manually; which is both time consuming and too complex for non-experienced users. To avoid this, we implemented a command procedure (cURL) [33] on the server side which forces GeoServer to publish the data automatically without going through GeoServer's web admin tool. Coding wise, this is simplified as both the projection and bounding box are constant for all the model outputs in this project and a default style can be chosen depending on whether the data is point or polygon in format. Once this process is completed the data is ready to be visualised and queried in the web viewer.

From a system operational standpoint, when the user selects a model for viewing, the aforementioned processes take place followed by the launching of the web viewer page. From here the system moves on to the orange pathways shown in Figure 2 to render the data and allow interaction.

### Display and Interact with data (orange path)

**Figure 3:**
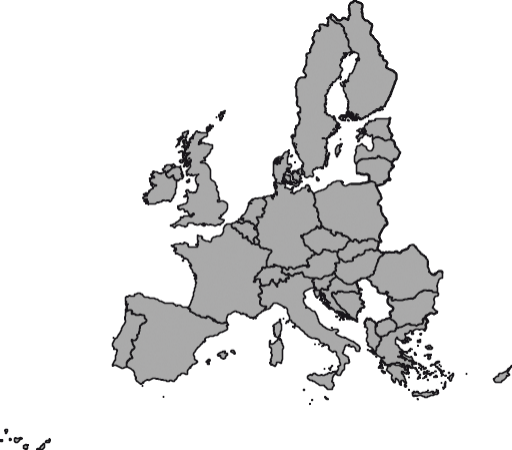
**Base map of Europe dataset**. This is an image of the base polygon map of the European dataset used in the examples within this paper.

Once the web page with the spatial visualisation tool has opened, a request is sent from the tool to GeoServer for the data to render in the map viewer. Data can be transmitted from server to user as a vector (via a Web Feature Service (WFS)) or as an image (via a Web Mapping Service (WMS)) as discussed previously. The amount of information required to be transmitted from server to the user and the level of possible interaction with the data is dependent on the format requested. For example, a comparison is made between the information transmitted from server to the user for rendering the base map used for the European models (Figure 3) as vector (WFS request) and as an image (WMS request) format. As a vector the amount of data that is transmitted from server to user is 3.26 MB. To transmit an image representation of the data (in a PNG format) consists of just 25 KB which is just 0.75% of the data transmitted in the WFS request. The reason why vector requests require more information to render is that spatial data for each vertex is required to represent the polygon. Taking the Republic of Ireland as a further example, the data required to represent all the vertices (held in the geometry column of the geodatabase) consists of a hexadecimal code comprising of 81,120 characters which equates to 80 kb.

Although WFS formats allow for greater levels of interaction on the user side, such as allowing for digitising and editing data; the WMS (image) formats can still be queried and interacted with to some degree as they are georeferenced by the map server. As the visualisation tool for this project was primarily designed for viewing and basic querying, a WMS approach for rendering data in the web viewer was selected. The WMS request renders a georeferenced image of the data in the web viewer which can then be interacted with.

**Figure 4:**
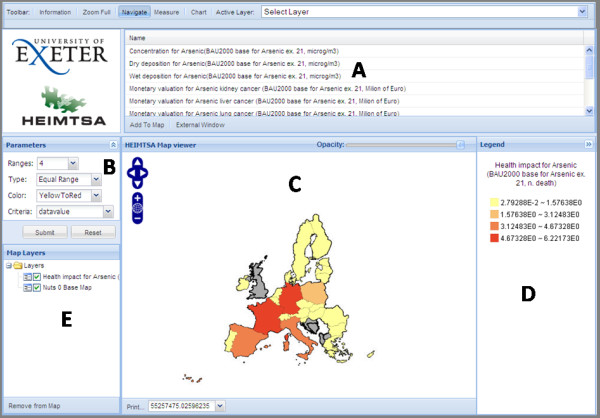
**Web Viewer Layout**. The web viewer interface is divided up into 5 regions labelled A to E. Region A contains list of available layers that can be added. Region B is the interface that allows for styling of selected layer. Region C is the map viewing panel where spatial data is displayed. Region D is the legend panel. Region E is the layertree where the order of the layers can be changed and where layers can be switched on and off.

The overall design of the web viewer consists of five key regions and a primary toolbar as shown in Figure 4. Regions A, B, C, and E are all areas where the user can interact with the data and region D provides information about the displayed data. The example data shown in region C is at NUTS 0 level - European wide country level data. We chose a panel based design, made via the use of the *GeoExt *and *Ext *JavaScript libraries. This overall design style was chosen due to its ease in implementation in creating toolbars, grids, and panel interfaces via the use of the script libraries and that it creates an intuitive and professional looking application. In addition, as the functionality of the program is embedded in the panels, the interface becomes portable and can be easily incorporated into any web site.

#### Adding layers (Region A)

It is envisioned that the users of this system may wish to compare multiple datasets from previous models within one session. The model list in this region is directly linked to the central geodatabase where all previous model output data is held. The models available in the list are unique to each user, as what is displayed in Region A is filtered according their login credentials.

#### Map viewing and interaction (Regions C & D)

**Figure 5:**
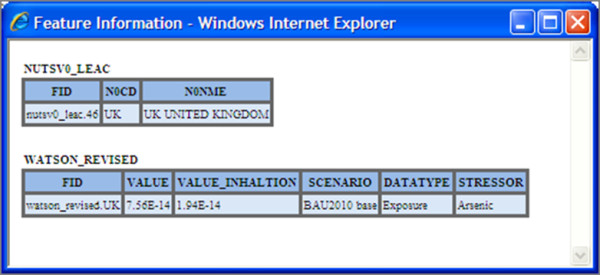
**Get feature information**. When a user selects a region within the map viewer via clicking on it with left mouse button this window appears with further information about the feature that was selected.

The map viewer is where the visualisation and interaction of data takes place. The client can pan and zoom either via the tool bar in the upper left hand corner of the viewer or by dragging the map and double clicking. One can also change the opacity of a layer via the use of a slider. If the user wants to query the data to get more attribute information, they can click on the map and get information about the layers at that location. This information is then displayed in a table format within a pop-up window shown in Figure 5 via a *GetFeatureInfo *request. The Layer Tree in region D allows the user to re-order the layers within the viewer and switch layers on and off.

#### Style data on the fly (Region B)

Being able to symbolise and customise the appearance of the data is one of the key features of this interactive visualisation tool; this allows the client to emphasise characteristics of their data. Symbolising geographical data can be complicated as the same data can be mapped as a choropleth, graduated symbol, dot, or surface/isopleth map ([34,35]). The choice of mapping method employed is related to the purpose of the map, the original data and how best to represent it. The choropleth map is a common mapping technique for areal data such as districts and counties. This technique is clearly appropriate when values of a phenomenon change abruptly at enumeration unit boundaries [35].

The basic styling of data within GeoServer is governed by a "Styled Layer Descriptor" (SLD) file; this is a static file which has to be written manually or via an external software package. The SLD file contains information about what symbols, colours and other attributes are associated with specific or a defined range of values.

For displaying results of a given study where the values of the outputted data are already known, referring to a predefined SLD file is possible, however, within this project the results are dynamically produced and thus unknown until a model has been completed. These results vary according to each model and therefore an adequate style associated with every dataset would not be possible. To solve this issue pre-existing code was used from an Open Source program that was developed as a dynamic thematic engine for GeoServer called *Geothematics *[36]. The Geothematics software provided the "on the fly" system that allows the re-symbolisation of the spatial data by the user. As the software was covered by the GNU General Public License it could be modified/tailored more specifically to our project's needs. *Geothematics *enables the automatic styling of data via four different class boundary classification methods shown in Table 2.

**Table 2:** Geothematics classification

Classification technique	Description
Equal Range	Divides the distribution of values (max - min) into equal ranges.

Equal Count	Creates intervals so that class each has an equal proportion of the sample.

Natural (Jenks) Breaks	Classes are defined according to apparently natural groupings of data values.

Standard Deviation	It shows the distance of an observation from the mean. It calculates the mean value and generates class breaks in standard deviation measures above and below it.

The Geothematics software allows for four methods of classifying data: Equal Range, Equal Count, Natural (Jenks) Breaks, and Standard Deviation.

**Figure 6:**
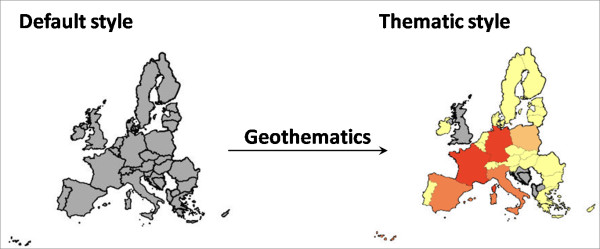
**Styling of data**. The Geothematic software generates a thematic map based on attribute values associated with each polygon, point, or line value.

The default style initially applied to data published by *GeoServer *assigns no colours to points or polygons by attribute. To create a choropleth map of the data a request is sent to the *Geothematics *SLD server to re-style the data by attribute. A simple equal range classification with just four classes is called by default as its quick to calculate and render; an example of before and after processing is shown in Figure 6. Further customisation/styling of a layer within the web viewer by the user is handled by the "Parameters" panel in region B (Figure 4). Here the user can specify the style of classification based on the four techniques mentioned previously and they can also choose the number of classifications, the colour scheme and what attribute from the data to base the style upon.

#### Additional Features

##### Visualising Uncertainty

**Figure 7:**
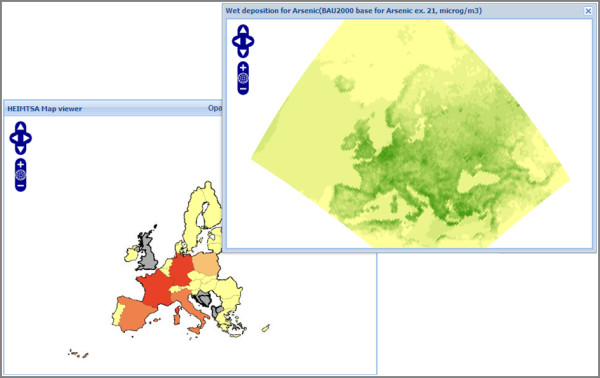
**Dual mapping**. The web GIS software allows for multiple linked maps to be opened simultaneously enabling cross comparison and for use with accompanying uncertainty data. This figure shows two linked maps open at the same time within the web viewer; one with polygon data and the other with point data.

Uncertainties are unavoidable when representing the real world because any model representation is incomplete [37] and the way that uncertainty is visualised is important in communicating details about the results and processes of a model [38]. There are a variety of methods for visualising uncertainty in spatial data, but one of the simplest is to use two adjacent maps, one of the data, the other of calculated uncertainties. This approach, to present model assessment and uncertainty in different maps is recommended as to avoid misinterpretation that could occur from information overload [39,40] if, for example, one overlaid uncertainties upon the result map. To enable two adjacent maps to be presented, the web viewer has been designed to enable uncertainty data to be viewed in an external floating window (Figure 7). Both maps are spatially linked with each other, using a linked-windows approach, therefore querying the location on one map yields the values for that location on both maps using the GetFeatureInfo response.

##### Graphical representations

**Figure 8:**
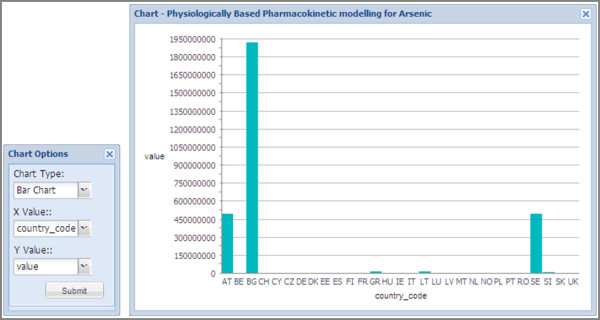
**Chart toolbar and resulting bar chart output for selected graph**. Using the chart toolbar function user can visualise data as a bar, line, or scatter chart. These charts can be visualised with the map data.

In addition to the spatial representation of data within the web viewer via the use of the *Ext *JavaScript libraries, it is possible to render chart based data in external windows within the web viewer. The user can select which attributes they wish to plot and choose between line, scatter, and bar charts, as shown in Figure 8.

If the user requires any assistance with the visualisation tool or wishes to find out details, a "Contact" button is included on the upper toolbar of the viewer. This will open a new window where the user can see development revisions. The client can also email the administrator comments and suggestions via the embedded message box. This is especially useful for continuing Open Source development.

### Overview of system operation

Once an external model is completed, and data is ready to be visualised, a button and a link become active on the associated web page viewed on the client side. When the user clicks on the button or link, an SQL code is generated and the process of publishing data (green path in Figure 2) begins. Upon completion of the publishing task the software launches the visualisation web page and opens the viewing tool whilst automatically switching to the "Display and interact" (orange paths) task to render the data. The model data that is to be rendered then needs to be styled so upon rendering a request is sent back to the *Geothematic *SLD server (red path) to style the data and re-draw in the web viewer. This complex network of functions is hidden from the user and the entire process occurs within a short timeframe. From a single click the user has gone from having tabular data into having a spatial representation of their data that they can now interact with.

## Discussion

### Development process

The development of this Open Source system was carried out over a nine month period. To achieve this within the short timeframe available, the functionality of the system was mainly limited to what were deemed "essential" features.

As the data being visualised by the system is potentially confidential or sensitive, restrictions on access were implemented. In order to access the external modelling tool, each user must first enter their login credentials (username and password). The Web visualisation tool described here has been designed so that the username filters the layers available to each individual user only.

### Value of development

The work undertaken has further highlighted the potential of using open source web GIS to enable the viewing and interrogation of environment and health data anywhere in the world freely via a simple web browser, either on a desktop or mobile device, as a viable alternative to commercial software. As it is open source development, and can thus be freely modified, it is hoped that with little modification this software could be used by others as a basis for allowing visualisation of their data online in a web browser, thus improving access to their data. This development acts like a technology equaliser, enabling economically restricted health and environmentally organisations, particularly in developing countries where the costs of implementing a system (e.g. for monitoring a disease outbreak, or dealing with waste removal from disaster relief camps) would be heavily restricted. One of the key features of this system is that no specialist software is required by the user on their computer. A low-end budget computer with internet access can use this software as the bulk of the system processes are all carried out on the server and not the client side. The front end panel based design created using *GeoExt *and *Ext *libraries means that the overall application is portable and can thus be embedded within any web page. This allows for the ease of distributing this application back to the open source community for others to use as a starting point and improve and develop further. By documenting the detailed development process we have followed in this paper, it is our hope that the problems and solutions we have implemented can further other future open source development.

### Example of application

Having described in detail and documented the development process in this paper, it is considered useful to add a brief example of the type of analysis that is likely to be conducted through this application. We take the example of the data shown in Figures 4 and 7, of Arsenic concentrations across Europe, and associated health impacts. Suppose a policy maker was interested in testing alternative scenarios to control arsenic emissions in an effort to reduce adverse health impacts (such as lung cancer) across Europe. One can model anticipated depositions using external models, but using our application, the results can be graphically visualised, explored and spatially data-mined. This adds an extra dimension to simply presenting results in a tabular or written form. We would see, for example as shown in Figure 4, that France and Germany have high potential health impacts due to arsenic contamination. We could drill down into either France or Germany to explore alternative attributes and map those, or alter class boundaries to re-visualise the country level data to more closely examine the spatial patterns. Using our second pop-up window to reveal uncertainties in our data, we would then be able to judge how confident we were in our modelled results. Based on these analyses, it is then the intention that the policy maker could make a more informed decision about what level of Arsenic concentrations are acceptable, what the health and monetary costs of these decisions are, and how they vary spatially across Europe.

### Future work

The spatial visualisation tool created for this project is now live and being hosted and used via the following website: http://heimtsa.jrc.ec.europa.eu/heimtsatb/. Users will have to register before accessing the system. Although the project initially set out to be a web based GIS, the level of functionality required lead the system to be more akin to a spatial data explorer/visualiser. Future development, for a different project, will expand the system as described here and expand the functionality to include greater analytical capabilities. Ongoing discussion with key public policy stakeholders will ensure that the system meets users' requirements and is thoroughly tested.

## Conclusions

The prototype visualisation tool developed here successfully enables users to spatially visualise model results in real time within a web browser, without the need for any additional software, or software training. The complex workings of the system are hidden from the user and the automatic rendering design used in this system enables users with no prior knowledge of GIS to visualise their data and immediately gain some understanding of the spatial structure of their data.

In comparison to that of commercial closed source software, open source is more complicated to initially implement. Although there is no specific dedicated customer support service, the support and advice provided by users in the open source community through forums and mailing lists is extensive, and there is a large community devoted to help and share ideas which can inspire "out of the box" thinking on solutions which may not be possible in closed source applications.

## Competing interests

The authors declare that they have no competing interests.

## Authors' contributions

Both authors contributed to the system design, co-wrote the manuscript and read and approved the final manuscript. BE is responsible for the coding. CES conceived the study, and participated in its design and coordination.
